# Physicians' Gender Influence on the Patients' Choice of Their Treating Obstetrician-Gynecologist in the Eastern Province of Saudi Arabia

**DOI:** 10.7759/cureus.23457

**Published:** 2022-03-24

**Authors:** Fatimah A Alsafar, Fehmida Tehsin, Kawther M Alsaffar, Walaa A Albukhaytan

**Affiliations:** 1 Medicine, King Faisal University, Al-Ahsa, SAU; 2 Obstetrics and Gynecology, King Faisal University, Al-Ahsa, SAU

**Keywords:** obstetrician-gynecologist, gender preference, patient care, female patient, saudi arabia

## Abstract

Introduction

Several studies have investigated the gender preference of obstetricians-gynecologists (OB-GYNs) around the globe. Obstetrics and gynecology deal with female patients exclusively, and gender selection in this special female domain bears significant cultural and religious aspects in childbirth and gynecological conditions.

Methods

This cross-sectional study has investigated women’s gender preferences of their OB-GYN in the Eastern Province of Saudi Arabia and assessed the factors that influenced their choice. A total of 390 female participants above 18 years of age answered a self-reported questionnaire distributed on different social media platforms from June 2021 to August 2021.

Results

A majority of the study participants have preferred female OB-GYN for pelvic examination, primary health screening, antenatal care, and major gynecological surgery (92.6%, 72.8%, 68.2%, and 61%, respectively). Almost one-third (31.3%) of the study females reported that they would allow the presence of a male obstetrician in the labor room. The highest three ranked factors affecting a patient's decision for choosing the gender of the OB-GYN were physician’s knowledge and experience, embarrassment with a male physician, and easiness to talk about women's issues with a female physician.

Conclusion

The vast majority of women in this study are inclined to choose female OB-GYN in general situations, for cesarean section, and in emergency situations.

## Introduction

Obstetrics and gynecology are essential domains of medicine. Almost all women visit a gynecologist during their lives [1], demonstrating the importance of this specialty. Nowadays, there are more female obstetrics and gynecology residents compared to male residents, making males doubtful to specialize in this field [2,3], therefore leading to less diversity and inadequacy among obstetricians-gynecologists (OB-GYNs) [4].

When patients are seeking medical care, they mostly lookup for a physician with good communication skills, surgical expertise, and a remarkable reputation, without having a certain gender preference if the health problem is not related to their genital organs. On the other hand, a physician’s gender plays a significant role when the health issue requires more private medical care. Female patients usually prefer a female physician in the discipline of obstetrics and gynecology [5] as well as in the urology domain [6]. Regarding the communication style, male physicians have a more directed approach, whereas female physicians possess a much more socioemotional and empathic communication approach characterized by nonverbal communication and higher expression of emotions [7].

Globally, several studies have reported and focused on this topic. Although most of the existing studies showed a female preference among the participants, a study, conducted in Turkey and published in 2014, showed that most of the participants (53.5%) had no preference regarding the gender of the OB-GYN [8]. On the other hand, a recent study, conducted in Pakistan and published in 2021, showed that the participants preferred female OB-GYNs and felt more comfortable with them [9]. These results are similar to those observed in a study conducted in the United States [10]. This preference should be taken seriously since some females would rather not go to a hospital that lacks female OB-GYNs. This may lead to complications as shown in a narrative review [11], where skipping the antenatal care visits among Syrian female refugees in Lebanon due to the lack of female OB-GYNs has resulted in higher rates of cesarean section among them. It is important to keep in mind the aspects that can improve the patient experience, suiting their needs [10].

Based on what we found, up to date, there are only five studies concerning the gender preference of OB-GYNs in Saudi Arabia. All of the five studies have shown very similar results. Three of them were conducted in Jeddah [12-14], one in Al-Khobar [15], and one in Al-Riyadh [5]. One study, conducted in Jeddah and published in 2021, showed that most of the participants preferred female OB-GYNs [14]. Interestingly, a recent study conducted in Jeddah has shown that 50.85% of the study participants expressed a strong preference for male OB-GYNs in emergency and critical surgery [13].

Considering the above, and since this issue is less commonly explored in the Eastern Province of Saudi Arabia, we sought to conduct this study aiming to investigate the women’s gender preferences for their OB-GYNs and the factors that influence their choice.

## Materials and methods

A cross-sectional study was conducted through a self-administered online survey, from June 2021 to August 2021, distributed among Saudi women in the Eastern Province of Saudi Arabia. Study objectives were explained, and confidentiality regarding the identity of participants was reassured by collecting filled questionnaires anonymously. Participants’ informed consent was taken before responding to the questionnaire. Study approval was taken from the Institutional Review Board in King Fahad Hospital (Hofuf), with research No. 09-EP-2021.

Saudi females, from the Eastern Province, who were 18 years and above were included in this study. Similarly, non-Saudi females, those from regions other than the Eastern Province, who were aged below 18 years were excluded from this study. A validated questionnaire from a previous study [9] was used with some modification and was translated from English to Arabic, the official language of Saudi Arabia. It takes approximately two to three minutes to complete the questionnaire, which is composed of 29 items in four sections. The first part was concerned with the sociodemographic data, including age, region, marital status, maternity, education level, occupation, income, and health insurance. The second part explored the participants’ previous experience with OB-GYNs. The third section was specified for women with previous visits to the OB-GYN department with questions regarding the subspecialty of the needed care, the usual gender of their OB-GYNs, and whether it was their own choice. Additional questions were concerned about the patient's satisfaction if they ever have changed the care provider because of the gender or have had any complications while receiving care. The fourth section was about the participants' gender preferences for OB-GYNs in general situations, emergencies, surgeries, and the factors influencing their choices.

At least 385 participants were required to be recruited for the study. We got a total of 460 responses, only 390 were eligible to be included in the study. The sample size was calculated using a sample size calculator with a 95% confidence interval and a 5% margin of error. We employed a convenience sampling technique.

After data extraction, data revision and coding were performed using the Statistical Package for the Social Sciences version 22 (SPSS, IBM Corp., Armonk, NY). Two-tailed tests were used for all statistical analyses. A statistically significant p-value was less than 0.05. Descriptive analysis based on the frequency and percent distribution was done for all variables including females’ sociodemographic data, maternity, children number, history of receiving obstetric and gynecology care, gender preference regarding different provided services by OB-GYNs, gender preference, and factors affecting the patients' choice of their treating OB-GYN. Cross tabulation was used to assess the distribution of OB-GYN gender preference by the biodemographic data of the females. Relations were tested using the Pearson chi-square test and the exact probability test for small frequency distributions.

## Results

A total of 390 eligible Saudi females completed the study questionnaire. The mean age of respondents was 28.9 ± 11.4 years. A vast majority (77.4%) of the females were married, and 20.3% were single. The majority (87.5%) had children, with variable parity status. More than half of the females (64.6%) were university students/graduates, and only 25.9% were employed. Regarding monthly income, 41.5% of females had 5000-10000 SR, while 29% had >10000-20000 SR income range. Exactly 46.9% of the study participants had got insurance (Table 1).

**Table 1:** Biodemographic data of participants

Biodemographic data	No.	%
Age in years		
18-25	147	37.7%
26-35	79	20.3%
36-45	87	22.3%
46+	77	19.7%
Marital status		
Single	79	20.3%
Married	302	77.4%
Divorced/Widowed	9	2.3%
Maternity (n = 312)		
I have children	273	87.5%
I don’t have children	39	12.5%
Children number (n = 273)		
One	51	18.7%
Two	65	23.8%
Three	53	19.4%
Four/more	104	38.1%
Educational level		
Pre-high school	4	1.0%
High school	83	21.3%
Diploma	51	13.1%
University/above	252	64.6%
Occupation		
Employed	101	25.9%
Unemployed	289	74.1%
Monthly income		
<5000 SR	68	17.4%
5000-10000 SR	162	41.5%
10000-20000 SR	113	29.0%
>20000 SR	47	12.1%
Insurance		
Yes	183	46.9%
No	207	53.1%

Table 2 illustrates the distribution of patients who received care in the obstetrics and gynecology department. A majority (80%) of the respondents had received care from an OB-GYN, and the most reported subspecialty of the needed care was General Obstetrics (77.6%) followed in descending frequency by General Gynecology (59%), Gynecological Oncology (7.1%), Urogynecology (5.1%), and Reproductive Endocrinology (2.6%). Most of the participants (92.6%) were treated by a female OB-GYN; 74.4% stated that it was their own choice. Almost 70.2% were highly satisfied regarding the provided care of the last seen OB-GYN, while 21.2% were moderately satisfied. Only 31.4% of the study females changed their treating OB-GYNs because of their gender. Among them, only a small number had encountered a complication, and a further lower percentage of them considered its relation with the gender of the treating OB-GYN.

**Table 2:** Distribution of patients who received care in the obstetrics and gynecology department OB-GYN: Obstetrician-gynecologist.

Patients who received care in the obstetrics and gynecology department	No.	%
Have you ever been provided care by an OB-GYN?		
Yes	312	80.0%
No	78	20.0%
What was the subspecialty of the needed care? (If more than one, choose all the applicable answers) (n = 312)		
General obstetrics	242	77.6%
General gynecology	184	59.0%
Gynecological oncology	22	7.1%
Urogynecology	16	5.1%
Reproductive endocrinology	8	2.6%
Others	0	0%
Don’t know	5	1.6%
What was the usual gender of your treating OB-GYN? (n = 312)		
Female	289	92.6%
Male	23	7.4%
Was it usually your choice? (n = 312)		
Yes	232	74.4%
No	80	25.6%
Gender of the OB-GYN you saw last time (n = 312)		
Female	272	87.2%
Male	40	12.8%
How satisfied were you with the care of the last seen OB-GYN? (n = 312)		
1-4	27	8.7%
5-7	66	21.2%
8-10	219	70.2%
Have you ever changed your treating OB-GYN just because of their gender? (n = 312)		
Yes	98	31.4%
No	214	68.6%
Was there any complication while receiving care? (n = 312)		
Yes	49	15.7%
No	263	84.3%
Do you think the gender of the treating OB-GYN played a role in the complication? (n = 47)		
Yes	8	17.0%
No	39	83.0%

Table 3 shows the distribution of gender preference among patients of their treating OB-GYN. The majority of the study participants preferred female physicians for pelvic exams (92.6%), primary health screening (72.8%), antenatal care (68.2%), and major gynecological surgery (61%). Moreover, 48.7% of the participants thought female physicians are better OB-GYNs. An exact 70% had no gender preferences regarding "which gender has more respect for their patients?", 69.7% had no preferences regarding "which gender has better bedside manners?", and 69.2% had no preferences regarding "which gender tends to spend more time with their patients?".

**Table 3:** Gender preference among patients of their treating OB-GYNs OB-GYN: Obstetrician-gynecologist.

Care service	Female		Male		No preference	
	No.	%	No.	%	No.	%
Who do you prefer for having primary health screening?	284	72.8%	12	3.1%	94	24.1%
Who do you prefer for pelvic exams?	361	92.6%	10	2.6%	19	4.9%
Who do you prefer for obstetric care of an unborn baby?	266	68.2%	21	5.4%	103	26.4%
Who do you prefer for major gynecological surgery?	238	61.0%	62	15.9%	90	23.1%
Which gender has more sympathy?	135	34.6%	76	19.5%	179	45.9%
Which gender is more trustworthy?	115	29.5%	59	15.1%	216	55.4%
Which gender has more respect for their patients?	49	12.6%	68	17.4%	273	70.0%
Which gender is more knowledgeable about women's health?	152	39.0%	34	8.7%	204	52.3%
Which gender has better bedside manners?	75	19.2%	43	11.0%	272	69.7%
Which gender tends to spend more time with their patients?	87	22.3%	33	8.5%	270	69.2%
Which gender is better OB-GYN?	190	48.7%	19	4.9%	181	46.4%

Table 4 reveals gender preferences and factors affecting the patients' choice of their treating OB-GYNs. Almost one-third (31.3%) of the study participants reported that they would allow the presence of a male OB-GYN in their labor room. Regarding factors affecting the decision for choosing a male or female OB-GYN, the three most reported factors with 61% were physician’s knowledge and experience, easiness to talk about women's issues with a female physician, and embarrassment with a male physician, followed by physician attitude and professionalism (45.6%), physician availability (29.2%), and female physician who is more patient and understanding (14.4%). Physician reputation was the least reported factor (3.3%); 45.4% of the study females preferred female OB-GYNs in emergency situations and in surgeries, while only 9.7% preferred male OB-GYNs. Also, 68.2% of the female respondents would get embarrassed when seeing a male OB-GYN, while 25.1% were equally embarrassed by both genders. As for the OB-GYN age, only 37.3% of the study participants preferred physicians older than them, while 61.8% stated that the age does not matter. When selecting an OB-GYN, the most important factor that mattered to the participants was those physicians who give full attention and not rushed (34.4%), followed by those who involve you in the treatment plan (20.3%), those who are easy to talk to about personal issues (17.2%), those who are easily accessible for appointment and question (11.5%), gender factor (7.9%), and sympathetic (5.6%), while 3.1% selected all of these factors.

**Table 4:** Gender preferences and factors affecting the patients' choice of their treating OB-GYNs OB-GYN: Obstetrician-gynecologist.

Factors	No.	%
Would you allow the presence of a male OB-GYN in your labor room?		
Yes	122	31.3%
No	268	68.7%
Factors affecting the decision for choosing a male or female OB-GYN (If more than one, choose all the applicable answers)		
Knowledge and experience	238	61.0%
Easy to talk about women's issues with a female physician	238	61.0%
Very embarrassed with a male physician	238	61.0%
Physician attitude and professionalism	178	45.6%
Physician availability	114	29.2%
A female physician is more patient and understanding	56	14.4%
A male physician is more patient and understanding	28	7.2%
A male physician is not affected by mood	28	7.2%
A male physician is more knowledgeable	23	5.9%
Physician reputation	13	3.3%
Others	14	3.6%
Religious factors	11	2.8%
Previous experience	1	.26%
Gender does not matter	1	.26%
Not specified	1	.26%
Who would you prefer in emergencies?		
Female OB-GYNs	177	45.4%
Male OB-GYNs	38	9.7%
Gender does not matter	175	44.9%
I’m embarrassed when seeing an OB-GYN if		
He was male	266	68.2%
She was female	1	.3%
Not embarrassed	25	6.4%
Equally embarrassed	98	25.1%
I prefer it when the OB-GYN is		
Older than me	147	37.7%
Younger than me	0	0%
Same age as me	2	.5%
Age does not matter	241	61.8%
When selecting an OB-GYN, what is the most important factor?		
Gives full attention and is not rushed	134	34.4%
Involves you in the treatment plan	79	20.3%
Easy to talk to about personal issues	67	17.2%
Easily accessible for appointment and question	45	11.5%
Gender	31	7.9%
Sympathetic	22	5.6%
All of the above	12	3.1%

Table 5 demonstrates determinants of preferred gender selection of the patients’ preferred gender as OB-GYN. Marital status and monthly income had an association with females’ gender preference. An exact 66.7% of the divorced/widowed females had no preference compared to 43.7% of the married group who mainly preferred female physicians (50.7% vs. 33.3%, respectively) with recorded statistical significance (p-value = .049). Also, 57.4% of females with higher income (>20000 SR) had no gender preference in comparison to 43.2% of those with lower income (5000-10000 SR) (p-value = .007).

**Table 5:** Determinants of preferred gender selection ^#^Exact probability test. ^*^P < .05 (significant). OB-GYN: Obstetrician-gynecologist.

Factors		Preferred gender as OB-GYN						P-value
		Female		Male		No preference		
		No.	%	No.	%	No preference	%	
Age in years	18-25	68	46.30%	5	3.40%	74	50.30%	.535^#^
	26-35	40	50.60%	4	5.10%	35	44.30%	
	36-45	45	51.70%	3	3.40%	39	44.80%	
	46+	37	48.10%	7	9.10%	33	42.90%	
Marital status	Single	34	43.00%	2	2.50%	43	54.40%	.049*^#^
	Married	153	50.70%	17	5.60%	132	43.70%	
	Divorced/Widowed	3	33.30%	0	0.00%	6	66.70%	
Maternity	I have children	139	51.10%	16	5.90%	117	43.00%	0.373
	I don’t have children	17	43.60%	1	2.60%	21	53.80%	
Children number	1	23	45.10%	2	3.90%	26	51.00%	.915^#^
	2	35	53.80%	4	6.20%	26	40.00%	
	3	27	50.90%	3	5.70%	23	43.40%	
	4+	55	52.90%	7	6.70%	42	40.40%	
Educational level	Pre-high school	3	75.00%	0	0.00%	1	25.00%	.688^#^
	High school	38	45.80%	6	7.20%	39	47.00%	
	Diploma	23	45.10%	1	2.00%	27	52.90%	
	University/above	126	50.00%	12	4.80%	114	45.20%	
Occupation	Employed	47	46.50%	7	6.90%	47	46.50%	0.517
	Unemployed	143	49.50%	12	4.20%	134	46.40%	
Monthly income	<5000 SR	33	48.50%	4	5.90%	31	45.60%	.007*
	5000-10000 SR	90	55.60%	2	1.20%	70	43.20%	
	>10000-20000 SR	48	42.50%	12	10.60%	53	46.90%	
	>20000 SR	19	40.40%	1	2.10%	27	57.40%	
Insurance	Yes	92	50.30%	9	4.90%	82	44.80%	0.834
	No	98	47.30%	10	4.80%	99	47.80%	
Have you ever had provided care from an OB-GYN?	Yes	155	49.70%	15	4.80%	142	45.50%	0.748
	No	35	44.90%	4	5.10%	39	50.00%	

## Discussion

The analysis of the study results indicates that the majority of the participants preferred female OB-GYNs for pelvic exams, primary health screening, and antenatal care with a percentage of 92.6%, 72.8%, and 68.2%, respectively. Similarly, in a recent cross-sectional study [5] of 3015 Saudi females residing in Riyadh, Alyahya et al. found that the majority of women preferred a female physician to perform a general examination (65.8%) and genital examination (86.4%). Female physicians were also preferred for conducting vaginal delivery in different studies [5,13].

A Saudi study by Shamrani [12] showed that most of the participants thought female OB-GYNs were better OB-GYNs than their male counterparts. Similarly, nearly half the percentage was of the same view in our study. Moreover, a majority of the participants preferred female OB-GYNs for major gynecological surgery. Similar results have been documented in two Saudi studies [12,14] conducted in Jeddah.

Another Saudi study [13], conducted also in Jeddah, showed that 46.64% of the participants preferred male OB-GYNs over females in cases of cesarian sections. Similarly, surgical skill was the main reason for choosing male OB-GYNs among 100 (14,2%) women in another study [8]. Although 44.9% of this study participants stated that gender does not matter in emergency situations, 45.4% of them preferred female OB-GYNs unlike the other study [13], where 50.84% of the study sample preferred male OB-GYNs in emergency and critical gynecological surgery.

Several studies around the globe have investigated the preferred gender of OB-GYNs. Similar to our study, a study done by McLean et al. [16] in 2012, in Al Ain, United Arab Emirates, has shown that 96.8% of the participants preferred female physicians in gynecological scenarios. In 2014, a Nigerian study [17] reported that 59.2% of the respondents preferred to be seen by female OB-GYN, although the majority were often seen by male OB-GYN. In the United States, two studies were concerned with OB-GYN’s genders. One of the two [18] was conducted in the Southeastern United States in 2013 and showed that 78% of the participants had a preference for female OB-GYN. The other one was a national survey [19] conducted in 2020 and showed that 66% of the participants preferred female OB-GYNs. On the other hand, in a cross-sectional study [8] conducted among Muslim women in Turkey, out of 710 women, 380 (53%) had no preference regarding the gender of their treating OB-GYNs.

Physicians' knowledge and experience, easiness to talk about women’s issues with female physicians, and embarrassment with male physicians were the highest selected factors by a majority of the respondents among the factors affecting the patients' choice of gender of their treating OB-GYNs. The physician attitude and professionalism (45.6%), physician availability (29.2%), and female physician who is more patient and understanding (14.4%) were found in descending order of percentages. When comparing these results to similar previous studies, two studies, conducted in Jeddah [12,14], showed that the most affecting factors were knowledge and experience, board certification, and recommendation by relatives or friends. Although reputation is one of the important factors found in those two studies, physician reputation was the least selected factor in our study. One of the studies [13] conducted in Jeddah showed that confidence in handling emergency situations, knowledge, and sympathy were the most influencing factors for their gender choice selection. Other two studies done in Pakistan and the United States [9,10] reported that the comfort feeling with a physician from the same gender is one of the important factors.

Additionally, our study revealed that a majority (68.2%) of the participants are embarrassed when being examined and seen by a male OB-GYN. Similarly, most of the previous studies reported a high percentage of embarrassment when being seen by a male OB-GYN. The surprising fact is that it is not just embarrassing for the patients but also for the doctors. A narrative review [11] showed that male OB-GYNs also would feel uncomfortable when treating females who have a cultural preference for female OB-GYNs.

In our study, one-third of the study participants reported that they would allow the presence of a male OB-GYN in their labor room, but the majority would not. This finding matches those observed in earlier studies. A qualitative study conducted in Khobar, Saudi Arabia [15], showed that three females feel uncomfortable while having a male OB-GYN in the labor room. Moreover, a narrative study [11] showed that 18 Somali refugee women in the United States also have the same idea but toward male medical students. These women felt like male medical students are generally unwelcomed. However, a systematic review and meta-analysis [20] done in 2017 with 23 studies showed that the majority (70.3%) of the participants would feel comfortable with either female or male medical students when they participate in the gynecology appointment.

Furthermore, this study has also explored the factors affecting the patients' choice of their treating OB-GYN. Regarding age, only one-third of the study participants preferred physicians older than them, while 61.8% stated that age does not matter. However, most of the study participants in the study by Shamrani [12] preferred OB-GYNs to be older than them.

Marital status and monthly income had an association with the females’ gender preferences. A majority of the divorced/widowed females had no gender preference, but on the other hand, a majority of the married females preferred female OB-GYNs. A possible explanation might be the role of the husband as 72% of the men participants preferred female OB-GYNs to deliver their female relatives in the study conducted by Alyahya et al. [5].

Regarding monthly income, more than half of the females with higher income (>20000 SR) had no gender preference in comparison to more than one-third of those with lower income (5000-10000 SR). This is totally different from other studies. One of them [14] showed that females with higher income preferred male OB-GYNs and specifically non-Saudi male OB-GYNs, while the other [12] showed that females with higher income preferred female OB-GYNs. In the study that was conducted among Muslim Turkish women [8], income level was the only factor that has a significant association (p-value < .05). Women with sufficient income tend to have no preference, and those with lower income have more preference toward female physicians.

The strength of this study is it's being one of the first studies carried out in the Eastern Province of Saudi Arabia that has explored the factors affecting the female patients' choice about the gender of their treating OB-GYNs. The response rate was good as the study touches the life of every woman. The limitations of this study involve its study design, and the results cannot be generalized across Saudi Arabia.

## Conclusions

The gender of the OB-GYN is an issue that needs to be addressed, especially in the context of Saudi culture. This study concludes that the majority of Saudi women in the Eastern Province tend to choose a female OB-GYN in general situations, cesarean section, and emergency conditions. The most chosen factors involved in this selection were physicians' knowledge and experience, easiness to talk with a female physician, and embarrassment with a male physician. Around seven out of 10 participants chose that they would not allow the presence of a male OB-GYN in their labor room. Current study findings are almost consistent with few studies conducted in Saudi Arabia to date. It is recommended to increase the number of female OB-GYNs to minimize the complications caused by the patients' refusal to be seen by male OB-GYNs.
